# Duodenocolic and Cholecystocolonic Fistula: A Case Report of an Unusual Presentation

**DOI:** 10.7759/cureus.56445

**Published:** 2024-03-19

**Authors:** Ozair Khan, Karamveer Singh, Nayana S Kumar, Navin Kumar, Somprakas Basu

**Affiliations:** 1 General Surgery, All India Institute of Medical Sciences, Rishikesh, Rishikesh, IND

**Keywords:** complications of laparoscopic cholecystectomy, cholecystocolonic fistula, duodenocolic fistula, thermal bowel injury, laparoscopic cholecystectomy

## Abstract

Laparoscopic cholecystectomy is the established standard of care for addressing symptomatic gallstones, typically representing a straightforward and uncomplicated surgical procedure. However, patients exhibiting variant anatomy or local inflammation can present challenges to the surgeon, potentially leading to complications. In this context, we present the case of a 55-year-old woman who underwent a laparoscopic cholecystectomy for symptomatic gallstone disease at a different medical facility. Postoperatively, she was diagnosed with a case of duodenocolic fistula and cholecystocolonic fistula. Conservative treatment ensued with intravenous antibiotic administration, as well as enteral and parenteral feeding. Diagnosing cholecystocolonic fistula before surgery proves challenging, even with modern diagnostic and imaging tools. Despite its significance, there is limited information in the literature regarding the management of this infrequent finding. The approach to diagnosis and management is elaborated upon in the case report.

## Introduction

Cholelithiasis is one of the most common surgical diseases encountered in day-to-day practice. Studies estimate an incidence of 10%-15%, with regional variation and around 20% of cholelithiasis being symptomatic. Laparoscopic cholecystectomy is the established standard for treating symptomatic cholelithiasis and is usually one of the first minimal access surgical procedures done or assisted by residents in training, with an estimated 750,000 procedures done in the United States in 2019 [1]. Like any surgical procedure, it is associated with complications. Bile duct injury is a well-recognized complication associated with this procedure. The nonbiliary injuries do occur with equally significant severity but tend to be underreported in the literature [2]. Nonbiliary complications of laparoscopic cholecystectomy can be either procedure-related or access-related.

Access-related injury pertains to a trocar or Veress needle injury, mostly reported in either transverse colon or cecum or great vessels. Procedure-related injuries are usually due to dissection and adhesiolysis or the use of electrocautery and are usually reported in the duodenum [3].

Cholecystoenteric fistulas (CEFs) are a rare complication of cholelithiasis. They were first described by Thomas Bartholin in 1654. Although their prevalence varies across several reports, they are estimated to affect 3%-5% of patients with cholelithiasis, most commonly occurring in patients with long-standing cholelithiasis. Most common among CEFs is cholecystoduodenal fistula comprising 60% of all CEFs, with cholecystocolonic (10%-20%), cholecystogastric (5%-10%), and choledochoduodenal (less than 5%) fistulas being less common [4,5].

The classical patient with a CEF is an elderly woman in the seventh or eighth decade of life, often with a known medical history of biliary disease and multiple medical comorbidities. The management of a CEF classically includes open surgery with cholecystectomy, excision of the fistula, enterolithotomy if an obstructing enteric stone is present, common bile duct exploration, operative cholangiography, and reconstruction of the penetrated organ, though increasingly minimal access techniques are being used to reduce morbidity and mortality.

Here, we present the case of a 55-year-old female with symptomatic cholelithiasis for which she was planned for laparoscopic cholecystectomy. Intraoperatively, she was diagnosed with dense adhesions and a cholecystocolonic fistula (CCF), for which she underwent subtotal fenestrating cholecystectomy with ligation of the CEF tract close to the gallbladder. In the postoperative period, she had persistent seropurulent discharge from the drain site, for which she was referred to our center. Typically arising as a late consequence of chronic gallstone disease, CCF is the second most prevalent CEF, following the cholecystoduodenal fistula. The incidence is approximately 0.1% among routine cholecystectomies. Here, we elaborate on our experience with an uncommon case of CCF presenting with a duodenocolic fistula.

## Case presentation

A 55-year-old woman with symptomatic gallstone disease status post-laparoscopic cholecystectomy performed seven days at another facility presented to the emergency department of our institute with complaints of pain in the upper right abdomen and persistent seropurulent output from a corrugated rubber drain. The primary operating surgeon was consulted telephonically to discuss the patient’s medical history, the findings from the operation, the surgical procedure itself, and the patient’s course at that facility.

We learned that she had recently undergone a difficult laparoscopic subtotal fenestrating cholecystectomy, during which a suspected CCF (approximately 3 cm in length) was ligated and divided. The ligation was done flush with the gallbladder using silk 3-0 sutures, and a corrugated rubber drain was placed in the gallbladder fossa. She was fine until the second day after the operation when she developed seropurulent discharge from the drain site and pain in her right upper abdomen. She was managed conservatively, and when her condition did not improve on conservative treatment, was transferred to our center on the seventh day after the operation for further management. At the presentation, the patient complained of right upper abdominal pain and discharge from the drain site. However, she was vitally stable and was able to pass both feces and flatus.

The investigations revealed an elevated total leucocyte count of 14,730/mm^3^ while the remaining blood investigations were within the usual range (Table 1).

**Table 1:** Laboratory values of the case. SGOT: serum glutamic-oxaloacetic transaminase; SGPT: serum glutamate-pyruvate transaminase; PT: prothrombin time; INR: international normalized ratio; TG: triglycerides; GGT: gamma-glutamyl transferase; LDL: low-density lipoprotein; HDL: high-density lipoprotein

Investigation	Admission	Discharge	Reference value
Hemoglobin	10.6	10.5	11.0–13.0 g%
Total leucocyte count	14,730	13,600	4,000–11,000/cc
Platelets	298,000	379,000	150,000–450,000/cc
Total bilirubin	0.39	-	0.3–1.2 mg/dL
Direct bilirubin	0.16	-	0.1–0.3 mg/dL
SGOT/SGPT	32/33	-	0–35 IU
Alkaline phosphate	181	-	30–120 IU
GGT	51	-	0–38 IU
Total protein	6.0	-	6.6–8.3
Serum albumin	2.8	-	3.5–5.2
Viral markers	NR	-	-
Urea	15	18	17–43
Creatinine	0.77	0.69	0.55–1.02
Sodium	140	135	136–146
Potassium	3.3	4.1	3.5–5.1
PT/INR	15.4/1.36	-	-
Total cholesterol	175	-	50–200
Serum TG	153	-	50–200
LDL	121	-	<50
HDL	14	-	>100

**Figure 1:**
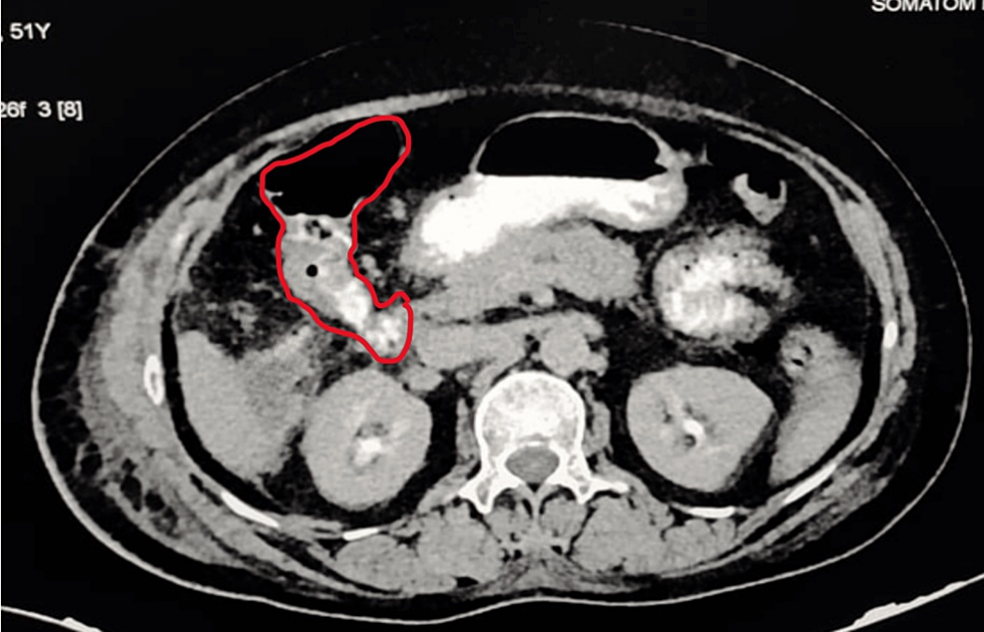
Contrast-enhanced scan showing contrast leaking from the transverse colon into the gallbladder fossa.

An abdominal contrast-enhanced CT scan was performed on the patient using oral and intravenous contrast, which revealed a collection measuring 2.6 x 2.3 x 2.3 cm in the gallbladder fossa (Figure 1). Air-fluid levels were observed in the collection. Upon administration of oral contrast, there was a suspicious communication between the second part of the duodenum and the hepatic flexure, as the contrast reached the transverse colon before fully opacifying the ileocecal junction and cecum. Additionally, a linear tract filled with contrast extending from the hepatic flexure to the gallbladder fossa was identified, measuring approximately 6.3 mm in length.

**Figure 2:**
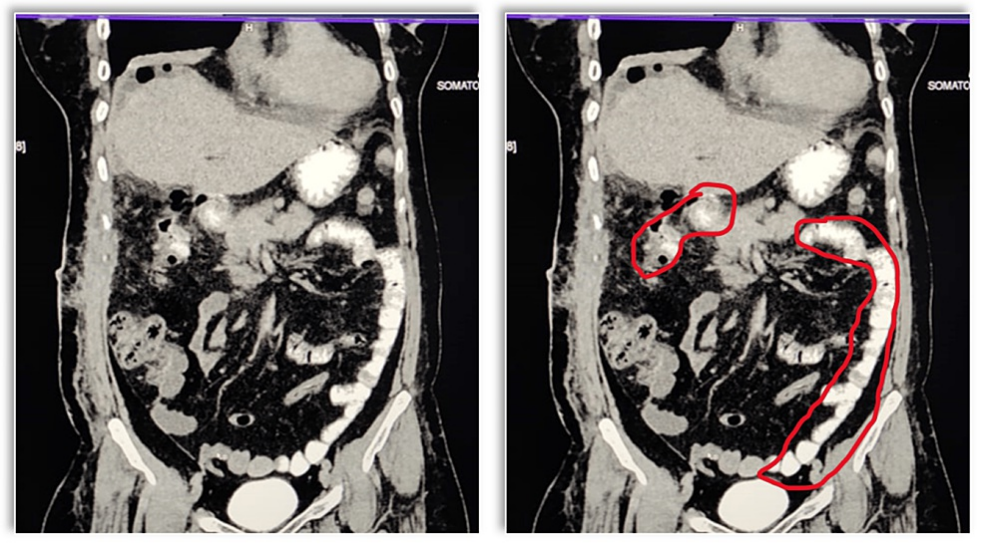
Contrast-enhanced scan showing contrast reaching the transverse colon and beyond, without opacification of the cecum and ascending colon, a suspected duodenocolic fistula. The encircled region in pane 2 shows the duodenum (marking on the top left corner) and transverse and descending colon (marking in the bottom right), respectively

Initially, she was administered intravenous broad-spectrum antibiotics and intravenous fluids and was initiated on parenteral nutrition. Subsequently, she was permitted to consume a high-protein liquid meal orally. The corrugated rubber drain was removed. A second contrast-enhanced CT of the abdomen was performed with rectal positive contrast and revealed a fistula tract connecting a collection in the gallbladder fossa to the hepatic flexure of the colon (Figure 2).

The patient experienced symptomatic improvement through conservative treatment. The output from the drain wound decreased to a minimal level, and the patient remained stable during their entire hospitalization. The drain wound developed healthy granulation tissue. She was gradually permitted to consume a soft diet, which she tolerated well. No underlying collection was observed on the repeat ultrasound of the abdomen which was done to look for collections. After seven days of conservative treatment, she was discharged from the facility in a stable state. On follow-up after 14 days, the patient was symptomatically better with a healthy wound, and a repeat ultrasound of the abdomen showed no underlying collections.

## Discussion

The rate of complications in laparoscopic cholecystectomy ranges from 3% to 7%, with intestinal injuries occurring in 0.07% to 0.7% of cases [6]. Courvoisier originally reported CEFs in 1890. The occurrence of these fistulas is primarily attributed to inflammation of the gallbladder caused by chronic cholecystitis [7]. CCF, affecting around 0.06%-0.14% of cases with biliary disease, is a rare and specific complication of gallstone disease. CCFs are usually found incidentally during cholecystectomy, with a rate of 0.5% in these procedures [8]. Failure to identify these fistulas during the surgery might result in significant consequences, such as unintentional separation of the fistula, perforation of the colon, the subsequent spread of fecal matter into the abdominal cavity, and peritonitis, which can lead to severe sepsis.

CCF commonly develops between the gallbladder and the hepatic flexure due to their proximity. Patients usually complain of general abdominal discomfort on presentation. The symptoms encompass diarrhea, stomach pain, jaundice, fever, nausea, vomiting, steatorrhea, and weight loss [9]; meanwhile, none of these symptoms were reported by our patient. Comparatively, fistulas that connect to the small intestine usually manifest as gallstone ileus. Pneumobilia, chronic diarrhea, and vitamin K malabsorption can be considered a specific triad that suggests the presence of a CCF (Savvidou et al.) [10]. None of these were found in our patient.

If a patient has a history of gallstones, cholecystitis, ascending cholangitis, gallstone ileus, obstructive jaundice, diverticulitis, or gastrointestinal cancer and is experiencing the symptoms indicated, a possibility of a fistula should be suspected. Preoperative studies have infrequently been able to make a definite diagnosis of CCF. Imaging may include ultrasound, CT, MRI, endoscopic retrograde cholangiopancreatography (ERCP), and barium enema, but a diagnosis is often made perioperatively [11]. When suspected intraoperatively, the diagnosis can be confirmed with a cholangiogram. However, ERCP has been considered the most accurate diagnostic modality of CCF preoperatively by some studies [12].

Traditionally, this condition has been addressed by performing fistula resection, cholecystectomy, and, if needed, common bile duct exploration and colonic resection. Segmental resection of the colon with anastomosis is typically advised when there is severe colonic inflammation or suspicion of cancer. Although an open method is usually preferred, multiple case reports have shown that laparoscopic approaches have comparable outcomes, though limitations of available expertise and longer operative periods remain. In our case, the patient underwent a subtotal laparoscopic cholecystectomy with ligation of CCF flush with the gallbladder and experienced increased drain output in the postoperative period, with cross-sectional imaging indicating a leak from the free end of CCF into the gallbladder fossa and a duodenocolic fistula. The patient was managed conservatively initially by keeping her nil per oral and starting empirical antibiotics, following which enteral feeds were resumed. Follow-up imaging demonstrated no residual collection and the patient was symptom-free on follow-up at two and four weeks. The leak from CCF was most probably caused by slippage of ligature tied during the initial procedure, while the mechanism of formation of duodenocolic fistula remains unclear. The patient was managed conservatively as she never developed signs and symptoms of sepsis or peritonitis, nor there was a free leak in the cross-sectional imaging.

## Conclusions

Here, we discussed a postoperative case of difficult laparoscopic cholecystectomy, wherein an intraoperative diagnosis of CCF was made and a decision of ligating tract flush with the gallbladder was taken, from which a leak was observed in the postoperative period on cross-sectional imaging, with clinical manifestation of increased drain output from postoperative day two. If a leak is detected in the postoperative period from CCF, close attention should be paid to the patient’s general condition. If the patient is vitally stable with so signs and symptoms of sepsis or peritonitis, conservative management can be done. All collections should be drained. If patients improve, they can be followed up with ultrasonography/cross-sectional imaging. Patients who fail to improve should be assessed, and if a free leak from the colon is noted, exploration and segmental resection should be done.

We conclude that all patients presenting with a CCF leak do not require exploration and can be managed conservatively if gross contamination and peritonitis are not present.

.
